# Supplementary material for: Terahertz waveform synthesis in integrated thin-film lithium niobate platform
Source: Nat Commun. 2023 Jan 4;14:11. doi: 10.1038/s41467-022-35517-6 (PMC9812977; doi:10.1038/s41467-022-35517-6)
Supplement: Supplementary file 1 — Supplementary Information [file 41467_2022_35517_MOESM1_ESM.pdf]

Supplementary information for

Terahertz waveform synthesis in integrated thin-film lithium niobate platform

A. Herter, A Shams-Ansari, F. F. Settembrini, H.K. Warner, M. Lončar, J. Faist, I.-C. Benea-Chelmus

Contents

Supplementary Note 1 (Sample) **3**

A Optical loss characterization . . . 3

B Waveguide modes . . . 3

C THz transmission through sample . . . 4

Supplementary Note 2 (Theory) **5**

A Optical rectification . . . 5

B Phase matching . . . 6

C Distributed pulse phase matching . . . 7

D Temporal mode decomposition . . . 8

Supplementary Note 3 (Simulation of waveguide propagation) **9**

A Current power configuration . . . 9

B High-power limit . . . 10

Supplementary Note 4 (Simulation of antenna structure) **11**

A Antenna size study . . . 12

B Gap length study . . . 14

C Control of THz polarization . . . 14

Supplementary Note 5 (Supporting Measurements) **15**

A Time-resolved THz electric field measurement . . . 15

B THz polarization . . . 16

C Coupling of optical signals . . . 16

D Efficiency of THz generation . . . 17

E Pump power dependency . . . 17

F Spatial map of cascaded antenna emission . . . 18

Supplementary Note 6 (Comparison with other terahertz generation schemes) **18**

Supplementary Note 7 (Increase of generated THz power) **20**

Supplementary Note 1 (Sample)

The fabrication process of our samples is explained in the methods section. The dimensions of the samples are listed in the Supplementary Table 1. For some antenna-parameters we give a certain range, since these values where changed for different investigations.

thickness of silicon substrate:	h_{Si}	500 µm
thickness of bottom silicon oxide layer:	$h_{\mathrm{SiO2,b}}$	2 µm
thickness of lithium niobate layer:	h_{LN}	300 nm
thickness of top silicon oxide layer:	$h_{\mathrm{SiO2,t}}$	800 nm
gold thickness:	h_{Au}	300 nm
length of bow-tie arm:	L_{ant}	40-200 µm
inner bow-tie arm width:	w_{ant}	5 µm
outer bow-tie arm width:	W_{ant}	30 µm
gap length:	l_{gap}	20-120 µm
antenna bar width:	w_{bar}	3 µm
antenna gap width:	w_{gap}	3 µm
width of lithium niobate waveguide:	w_{wg}	1.5 µm
waveguide height:	h_{wg}	600 µm

Supplementary Table 1: List of sample and antenna design parameters.

A Optical loss characterization

The minimum tolerable gap between the gold nano-antennas and the optical waveguides sets the maximum efficiency of the THz generation process. To determine the minimum allowable antennae gap, identical optical waveguides were fabricated with varying electrode separations and lengths. Optical absorption loss, shown in Supplementary Fig. 1 was characterized using an optical transmission measurement with -3.9 dBm on chip optical power at 1580 nm. Light is coupled into the system from a fiber grating array via grating couplers and transmission is normalized to bare waveguide transmission to extract loss figures and determine separation at which absorption in gold becomes the dominating loss mechanism. Gap separations below 3.6 µm were recorded at the noise floor of our detector. In order to estimate absorption in gold for gap separations below $w_{\mathrm{gap}} = 3.3\,\mu\mathrm{m}$, we fit the absorption to a decaying exponential and estimate that $w_{\mathrm{gap}} = 3\,\mu\mathrm{m}$ corresponds to expected $0.727\,\mathrm{dB\,cm^{-1}}$ loss.

B Waveguide modes

We simulated group refractive index for near-infrared signal inside the waveguide using CST microwave studio. In this case two waveguide ports are placed at the front and back surface of a short waveguide piece using the dimensions shown in Supplementary Table 1. The near-infrared field is mainly confined inside the LN waveguide and the simulations confirm the low interaction between gold and near-infrared signal for the chosen gap width $w_{\mathrm{gap}} = 3\,\mu\mathrm{m}$, so that the waveguide mode is similar for both cases (Supplementary Fig. 2 a and b). The group refractive index in the near-infrared determined from simulations are $n_{\mathrm{g}} = 2.337$ for the bare waveguide and $n_{\mathrm{g}} = 2.343$ inside the gap. The THz field along the gap for a discrete port is determined from the simulation of an antenna with a resonance frequency of 385 GHz (Supplementary Fig. 2 c).

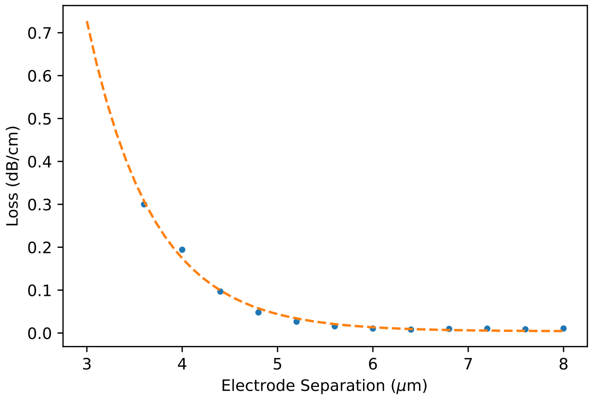

Supplementary Fig. 1: Optical loss of waveguides fitted with electrodes. Measured (blue) optical loss in 1.5 μm lithium niobate waveguide due to absorption in gold nano-antennae were fit to an exponential (orange) to estimate optical absorption for narrow antennae gaps.

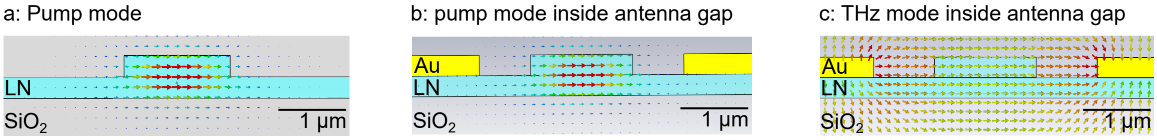

Supplementary Fig. 2: Near-infrared and terahertz modes. a and **b**: near-infrared pump field inside the bare waveguide (a) and within the antenna gap (b) simulated with CST Microwave Studio. **c**: THz mode inside the waveguide simulated in CST Microwave Studio.

C THz transmission through sample

One of the disadvantages of bulk lithium niobate for THz is the high absorption of the generated field. In our case the lithium niobate layer measures 600 nm, much shorter than the terahertz wavelength, so we assume the losses to be negligible. We measured the transmission of broadband THz signal through the fabricated TFLN wafer at a location without antenna structures to extract the transmission losses utilizing a commercial time-domain spectroscopy system provided by MenloSystems. The time-trace transmitted through the sample is compared to a reference measurement performed without the sample (supplementary Fig. 3 a). The signal transmitted through the wafer exhibits a second pulse after the main pulse, caused by reflection at the sample surface. The time distance of $\delta t = \frac{2n_{\mathrm{Si}}D}{c} = 11.4\,\mathrm{ps}$ between the two pulses is related to the sample thickness $D = 500\,\mu\mathrm{m}$ and its refractive index $n_{\mathrm{Si}} = 3.425$ [1]. In case of THz signals shorter than 11.4 ps, we observe a second pulse in the measurements of our on-chip antenna emitters (compare Fig. 2 a in the main text) with a similar time-delay, which is also explained by reflection. To investigate the frequency dependent losses due to reflection and absorption the Fourier transformation of the main peak giving the spectral electrical field is compared to reference measurement (Supplementary Fig. 3 b). The bandwidth of the utilized THz source reaches up to more than 1.5 THz, which is larger than the frequency range covered by the measurements in this work. The ratio between the field transmitted through the sample without being reflected and the reference field shows a flat behavior up to 1 THz (Supplementary Fig. 3 c). Considering the reflection at two silicon-air-interfaces described by Fresnel equations, the expected ratio between input and transmitted field measures

$$\frac{E_{\mathrm{t}}}{E_{\mathrm{in}}} = (1 - r^2) = \frac{4 \cdot n_{\mathrm{Si}}}{(1 + n_{\mathrm{Si}})^2} = 0.70. \qquad (1)$$

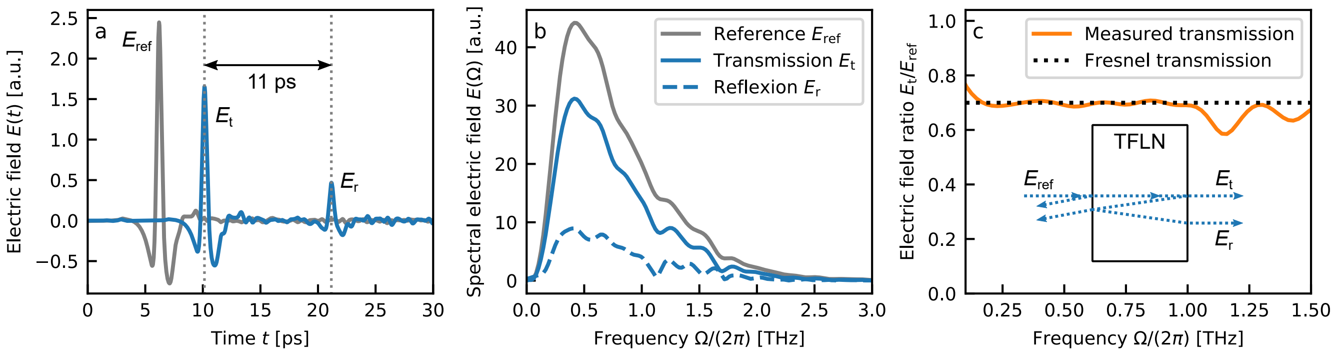

Supplementary Fig. 3: THz tranmission through TFLN wafer stack. **a**: Time-trace of the transmission through the thin-film lithium niobate chip (blue) compared to the emission of the utilized THz source (grey). **b**: Comparison of the spectral electric fields of the main peak with the reference measurement and the reflected signal in a. **c**: Measured ratio between incoming and transmitted THz spectral field compared to the value expected from Fresnel equations.

The determined value (dashed line in Supplementary Fig. 3 c) and matches the measured coefficient, indicating that all losses can be explained by reflection at the sample surfaces. The layer of high-absorbing lithium niobate does not effect the transmitted field noticeably due to the thickness of only 600 nm (300 nm after etching the ridges). Consequently the THz radiation generated inside the waveguides can leave the lithium niobate without significant absorption losses. Given that the propagation loss of the terahertz radiation inside the lithium niobate layer is negligible, we presume that cooling the chip may not lead to significant increase in the emitted terahertz field amplitude.

Supplementary Note 2 (Theory)

A Optical rectification

Based on the theoretical description of optical rectification in bulk nonlinear crystals [2] we will describe the electric field generated inside the antenna gap and conclude on the expected spectral characteristics of signal emitted by our on-chip THz emitters. In general, the nonlinear polarization introduced by optical rectification

$$P_{\mathrm{OR}}(\Omega) = \frac{\chi^{\mathrm{OR}}(\Omega,\omega_0)}{n(\omega_0)\cdot c}\cdot I_0(\Omega) \tag{2}$$

is determined by the intensity envelope of the optical pump pulse $I_0(\Omega) = \mathcal{FT}(I_0(t))$ propagating in y-direction, the second order susceptibility causing optical rectification $\chi^{\mathrm{OR}}(\Omega,\omega_0)$ and the refractive index of the interaction medium at the center frequency of the pump $n(\omega_0)$. By solving the nonlinear wave equation in a plane wave approximation Schneider et al. [2] derives the electric THz field generated in a bulk crystal after a propagation length y as:

$$E_{\mathrm{bulk}}(\Omega,y) = C_{\mathrm{bulk}}\cdot I_0(\Omega)\cdot\Omega\cdot\frac{\exp\left(-i\frac{\Omega n(\Omega)}{c}y\right)-\exp\left(-i\frac{\Omega n_{\mathrm{g}}}{c}y\right)}{i\cdot\frac{\Omega}{c}\cdot(n(\Omega)-n_{\mathrm{g}})}, \tag{3}$$

where the absorption of the both, the THz and the near-infrared, are neglected. $C_{\mathrm{bulk}} = \frac{\mu_0\cdot\chi^{\mathrm{OR}}(\Omega;\omega_0)}{i\cdot n(\omega_0)\cdot(n(\Omega)+n_{\mathrm{g}})}$ is a constant term that depends on the vacuum permeability μ_0, the second order nonlinear susceptibility causing optical rectification $\chi^{\mathrm{OR}}(\Omega;\omega_0)$, the refractive index at the THz frequency $n(\Omega)$, at the pump frequency $n(\omega_0)$ and the group refractive index of the pump pulses n_{g}. The term $I_0(\Omega)\cdot\Omega$ includes the spectral behavior of optical rectification and the fraction describes the phase-matching between of the THz and pump field along the generation region. If

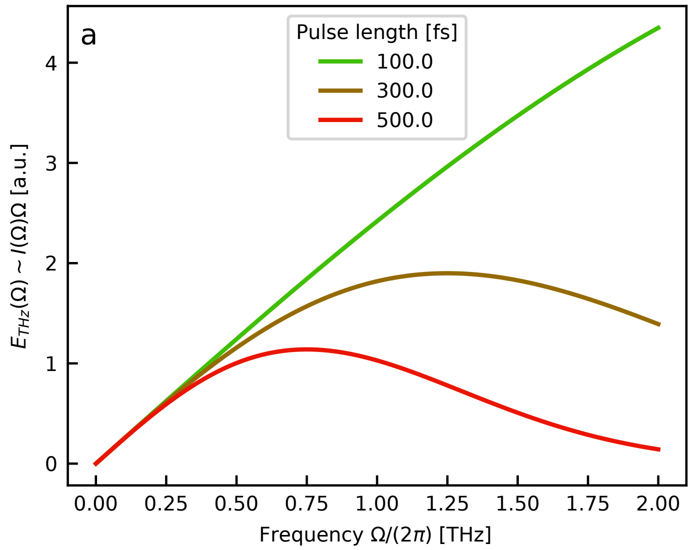

Supplementary Fig. 4: Calculated THz spectrum generated by a Gaussian pump pulse of varying pulse length, but constant pulse energy.

only interested in the amplitude of the generated spectral field, the latter can be expressed by a frequency dependent effective generation length $l_{\text{eff}}(\Omega, y)$ defined as the absolute value of the phase-matching term:

$$l_{\text{eff}}(\Omega, y) = \left| \frac{\exp\left(-i\frac{\Omega n(\Omega)}{c} y\right) - \exp\left(-i\frac{\Omega n_{\text{g}}}{c} y\right)}{i \cdot \frac{\Omega}{c} \cdot (n(\Omega) - n_{\text{g}})} \right|. \tag{4}$$

For small generation lengths below the coherence length $l_{\text{coh}} = \frac{c}{4 n_{\text{g}} f_{\text{THz}}}$ the spectral properties of the generated field are mainly determined by the pulse length t_{FWHM} of the near infrared signal. For a Gaussian pulse with a pulse length $t_{\text{FWHM}} = 2\sqrt{2\ln 2}\,\tau$, pulse energy E_{pulse} and effective mode size A_{mode}, $I(\Omega)$ is given by

$$I(\Omega) = \frac{E_{\text{pulse}}}{A_{\text{mode}}\sqrt{2\pi}} \exp\left(-\frac{\tau^2\Omega^2}{2}\right). \tag{5}$$

As shown in Supplementary Fig. 4 a, E_{THz} increases linearly with frequency in the low spectral range independently from the pump pulse length. The latter determines the upper frequency limit. With increasing pulse length the deviation from the linear increase shifts towards lower frequencies. The slope of the increase is determined by the pulse energy E_{pulse} per mode size A_{mode}.

We assume that the spectral behavior of optical rectification inside an antenna gap is mainly determined by $|E_{\text{OR}}| \sim |I_0(\Omega)| \cdot \Omega \cdot l_{\text{eff}}(\Omega, l_{\text{gap}})$ as well. Strictly speaking, for the case of optical rectification inside a metallic gap of sub-wavelength dimensions, the description by a 1D nonlinear wave-equation and the plane-wave approximation as in [2] does not fully apply anymore.

B *Phase matching*

The THz field is detected perpendicular to the chip surface. The locally generated field excites the antenna, and the phase-delay between the different sources along the antenna gap is mainly determined by the propagation speed of the pump pulse $v_{\text{g}} = \frac{c}{n_{\text{g}}}$. We neglected the influence of the THz signals propagating along the antenna because of their very small intensities. Consequently the high absorption of lithium niobate does not significantly affect the performance of our emitters. Losses occurring while propagating through the substrate are constant for all local fields along the generation area, so that they only affect the constant factor C_{gap}. The overall electric field exciting the

THz antenna E_{OR} is derived by integrating the locally induced electric field $E_{\mathrm{local}} \sim I_0(\Omega) \cdot \Omega \cdot \exp\left(-i \frac{n_{\mathrm{g}}\Omega}{c} y\right)$ along the antenna gap in y-direction:

$$E_{\mathrm{OR}}(\Omega) = C_{\mathrm{gap}} \cdot I_0(\Omega) \cdot \Omega \cdot \int_0^{l_{\mathrm{gap}}} \exp\left(-i \frac{n_{\mathrm{g}}\Omega}{c} y\right) \mathrm{d}y \tag{6}$$

$$= C_{\mathrm{gap}} \cdot I_0(\Omega) \cdot \Omega \cdot (-i) \cdot l_{\mathrm{gap}} \cdot \exp\left(-i \frac{n_{\mathrm{g}}\Omega}{2c} l_{\mathrm{gap}}\right) \cdot \mathrm{sinc}\left(\frac{n_{\mathrm{g}}\Omega}{2c} l_{\mathrm{gap}}\right) \tag{7}$$

$$|E_{\mathrm{OR}}(\Omega)| = C_{\mathrm{gap}} \cdot |I_0(\Omega)| \cdot \Omega \cdot l_{\mathrm{eff}}(\Omega). \tag{8}$$

The integration over the phase-factor $\exp\left(-i \frac{n_{\mathrm{g}} z}{c} \Omega\right)$ is giving the effective generation length $l_{\mathrm{eff}}(\Omega)$ for the on-chip optical rectification. For a gap length $l_{\mathrm{gap}} \leq \frac{2\pi c}{4 n_{\mathrm{g}} \Omega} = l_{\mathrm{coh}}$ – as it is the case for all investigated antenna designs – the local fields will sum up constructively, so that the influence of phase matching is rather small. Consequently the spectrum generated inside the antenna gap is mainly determined by the temporal shape of the pump pulse described by the spectral intensity envelope $I_0(\Omega)$. For low frequencies the spectral electric THz field grows linear with the intensity, while the upper limit of the spectrum is mainly limited by the pulse length of the pump signal, which determines the spectral width of its envelope $I_0(\Omega)$.

C Distributed pulse phase matching

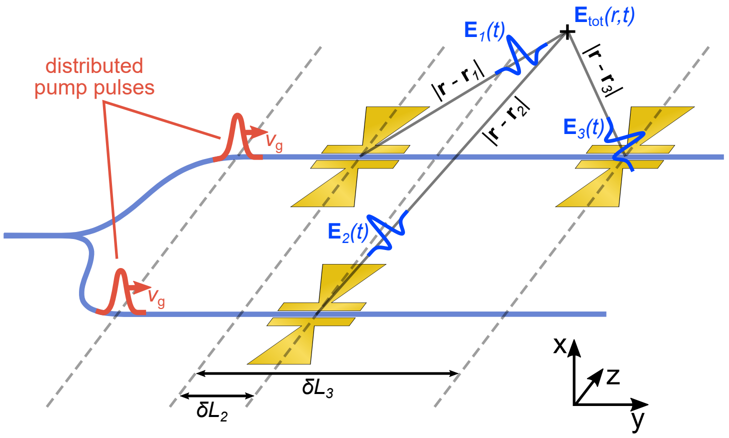

Supplementary Fig. 5: Schematic illustration of the phase-delay between THz pulses originating from different gold antennas at positions $\mathbf{r}_i$. The generating pump pulses are distributed in different device arms using a waveguide splitter. The relative distances of the antennas to the first one δL_i determine the time-delay between the respective generation progresses due to the finite propagation speed of the pump signal v_{g}. Additionally the THz signals travel different distances $|\mathbf{r} - \mathbf{r}_i|$ before they overlap in the particular observation point $\mathbf{r}$ resulting in a certain phase-shift at this position.

To describe the overall field emitted by a device $\mathbf{E}_{\mathrm{tot}}$, the electric fields generated by the contributed antennas $\mathbf{E}_i$ are summed up, whereby the temporal and spectral properties of $\mathbf{E}_i$ is determined by particular antenna design. The position of the antennas on the chip r_i as well as the waveguide distance between them δL_i lead to a phase-shift of the THz pulses (Supplementary Fig. 5). Therefore we describe the total field observed at a position $\mathbf{r}$ as the sum the contributed signals including a retardation time:

$$\mathbf{E}_{\mathrm{tot}}(\mathbf{r}, t) = \sum_i \mathbf{E}_i \left(t - \frac{\delta L_i}{v_g} - \frac{|\mathbf{r} - \mathbf{r}_i|}{c_0}\right). \tag{9}$$

The first correction term corresponds to the time delay between antennas being triggered by the pump pulse due to the difference in propagation length δL_i inside the waveguides. Additionally the THz signals travel different distances

$|\mathbf{r}-\mathbf{r}_i|$ through the free space until they arrive at the point of observation. Therefore the choice of antenna design in combination of their arrangement on chip and the waveguide path enables to custom-tailor the frequency-components, their phase-delay and the farfield shape of the emitted radiation.

D Temporal mode decomposition

In this section, we provide a theoretical model that allows to decompose an arbitrary terahertz field into a superposition of orthogonal temporal modes. Then, we show how the various free parameters of this mathematical decomposition can be directly mapped onto physical parameters of a fabricated TFLN terahertz waveform synthesizer that uses the basic blocks discussed in the main manuscript. We show that following this approach, a library of terahertz emitters may be generated to create the necessary basis of temporal modes. A careful arrangement of the terahertz emitters on-chip can then further be employed to synthesise any desired waveform.

In general, one can describe an arbitrary terahertz electric field that propagates in x-direction through its Fourier decomposition into coherent components that oscillate at frequencies ω:

$$\mathbf{E}_{\mathrm{THz}}(\mathbf{r},t) = \int d\omega \mathbf{E}(\mathbf{r},\omega) e^{-i\omega(t-\frac{|\mathbf{r}|}{c})}. \tag{10}$$

To ease the notation and without loss of generality, we will consider in the following only plane waves of a single polarisation. This allows us to reduce the description to

$$E_{\mathrm{THz}}(x,t) = \int d\omega E(\omega) e^{-i\omega(t-\frac{x}{c})}, \tag{11}$$

where c is the speed of light and $E(\omega)$ are the complex amplitudes of the individual coherent components.

We show in the main manuscript that single antennas generally emit broadband single- or few-cycle terahertz pulses around one particular center frequency, depending on their exact geometric dimensions. As a result, a decomposition of the total desired terahertz waveform into short temporal modes is better suited than into coherent modes at narrow-band frequencies as done above. Consequently, we now introduce here a different decomposition into a sum of discrete temporal modes described by an orthonormal set of complex envelope function $f_i(\omega)$ [3, 4], with a characteristic center frequency $\omega_{\mathrm{THz,k}}$, a carrier envelope offset phase $\phi_{i,k}$ and a group delay $\tau_{i,k}$:

$$E_{\mathrm{THz}}(x,t) = \sum_i \sum_k b_{i,k} \int d\omega f_i(\omega - \omega_{\mathrm{THz,k}}) e^{-i\omega(t-\tau_{i,k}-\frac{x}{c})-i\phi_{i,k}}. \tag{12}$$

All complex envelope functions are defined around $\omega = 0$, and fulfil the orthonormality and completeness relations

$$\int d\omega f_i^*(\omega) f_j(\omega) = \delta_{\mathrm{ij}} \tag{13}$$

$$\sum_i f_i^*(\omega) f_i(\omega') = \delta(\omega - \omega'). \tag{14}$$

In practice, we will also assume that the envelope functions are relatively narrow-band in the frequency domain, characterized by a linewidth Γ:

$$\int d\omega f_i^*(\omega) f_i(\omega') = 0, |\omega - \omega'| \geq \Gamma + \Gamma'. \tag{15}$$

As a results of these conditions, it becomes possible to construct an orthonormal basis of envelope functions $f_i(\omega)$ that govern the complex envelope of the terahertz signals, but also allow another dimensionality in frequency space, characterized by the center frequency $\omega_{\mathrm{THz,k}}$. We can choose for example Laguerre-Gaussian modes in frequency domain as the basis for $f_i(\omega)$, as an alternative to otherwise suggested Hermite-Gaussian modes in ref. [4].

In a more rigorous approach, a Gran-Schmidt orthogonalisation procedure may be performed on the discrete basis of frequency-dependent Laguerre-Gaussian modes, e.g. on all fundamental Laguerre-Gaussian modes at all considered center frequencies $f_0(\omega - \omega_{\mathrm{THz,k}})$, $\forall k$. For simplicity, we do not pursue this possibility further in our treatment.

Now that we have defined the orthogonal set of modes $\{i, k\}$ that are required for the decomposition, the only remaining question is whether it is - in principle - possible to control all the free parameters of equation (12) by chip-scale design alone. In the main manuscript, we show that the amplitude of the generated terahertz pulse can be engineered by the length of l_{gap}. This gives us a way to selectively set all amplitudes $b_{i,k}$. In addition, we have seen that the center emission frequency of an antenna $\omega_{\mathrm{THz,k}}$ can be set by the arm length. The group delay of an emission $\tau_{i,k}$ can be influenced by the length of the LN waveguide prior to the THz antenna. The linewidth of the antenna can be also influenced by the antenna design (e.g. LC-resonator vs bow-tie antenna). Finally, the carrier envelope phase of an antenna can also be set by antenna design.

Supplementary Note 3 (Simulation of waveguide propagation)

A Current power configuration

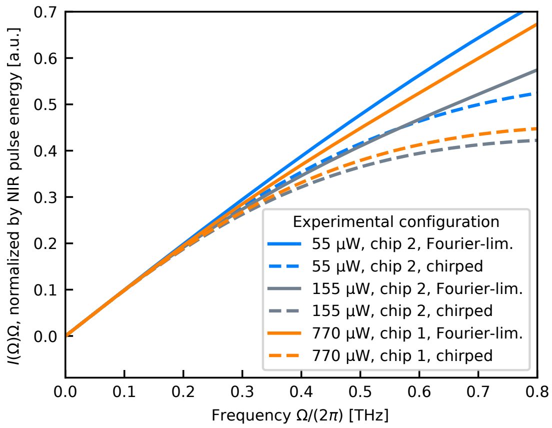

Supplementary Fig. 6: Calculated THz spectrum assuming Fourier limited (solid line) and chirped (dashed line) pump pulses based on the spectra measured in different experimental configurations (shown in Supplementary Note 5 C) after propagating through the TFLN chip.

In Supplementary Note 2 A we have shown the THz spectrum generated by optical rectification depends on the pulse shape of the NIR pump signal. To understand how the spectral and temporal changes inside the fiber and waveguide can affect the optical rectification process we now calculate the generated THz spectrum based on the pump spectra presented in the supplementary material figure 10 b and c for two simplified cases. First assuming the pump pulses being Fourier-limited (solid line in Supplementary Fig. 4 b) and secondly including a group delay dispersion of -0.02 ps^2 (dashed line in Supplementary Fig. 4 b) corresponding to the propagation in 1.1 m of optical fiber (GVD $= -19.65\mathrm{ps}^2/\mathrm{km}$) and 5 mm of TFLN waveguide (GVD $= 280\,\mathrm{ps}^2/\mathrm{km}$, determined for particular waveguide design with COMSOL Multiphysics Software). The phase shift caused by nonlinear effects inside the optical fiber and waveguide strongly depend on the pulse energy but will counteract the negative group delay dispersion. As a consequent, the true pulse length will be in between those two simplified cases.

Below 400 GHz the calculated spectral THz field is barely influenced by the different spectra or the considered chirp (Supplementary Fig. 4). For higher frequencies we observe a flattening of the THz field generated by the chirped pulses, while the spectral differences still do not influence the efficiency of the THz generation in a crucial manner.

In our specific experimental conditions, most of the investigated devices emit frequencies below 400 GHz. Therefore, the dispersion and nonlinear effects do not influence the THz signal significantly. For the study of the different antenna sizes, the spectral and temporal changes inside the fiber and TFLN waveguide have to be taken into account, since we reach frequencies up to 680 GHz. For this study we decreased the pump power to exclude the influence of nonlinear effects in both - the fiber and waveguide - onto our measurement results. For simulations we are comparing to the measured results, nonlinear effects will not be taken into account.

B High-power limit

While for the current study comparably low pump pulse energy of up to 100 pJ are utilized, the question arises as to how much we can increase the pump power in the future - especially to improve the generation efficiency and the emitted THz intensity. The nonlinear effects occurring inside the fiber coupling the laser signal to the TFLN chip can in principle be mitigated using free-space propagation of the pump light prior to the chip in combination to edge coupling. On contrary, the lithium niobate waveguides are an elementary part of our THz generation technique. Consequently, nonlinear effects inside the TFLN chip will limit the maximal power we can use for the generation process. This aspect is investigated based on simulation in the following.

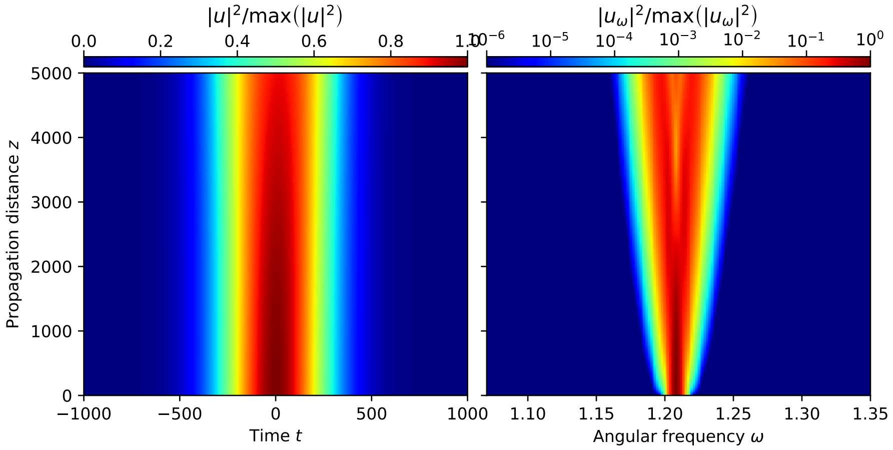

Supplementary Fig. 7: Simulation of a 500 fs-long Gaussian pulse with a pulse energy of 1 nJ in a 5 mm long TFLN waveguide performed with the python package fmas [5]. Left: Evolution of the temporal pulse-shape. Right: Signal evolution in the spectral domain.

We use the python package fmas [5] that solves the unidirectional nonlinear Schrodinger equation to simulate the pulse propagation inside the TFLN waveguides. The total dispersion is calculated starting from the effective mode refractive index that we simulated using the COMSOL Multiphysics Software. We assume a Kerr nonlinear refractive index to be the one of bulk LN: $n_2 = 1.8 \cdot 10^{-19}\,\mathrm{m^2/W}$ [6]. Furthermore, Raman scattering is expected to occur in lithium niobate. Here, we chose values of $f_R = 0.635$ for the Raman fraction, a period time of $\tau_1 = 21\,\mathrm{fs}$ and a decay of $\tau_2 = 544\,\mathrm{fs}$ in our simulation [7]. We note that the different values for f_R reported in the literature cause uncertainty in our simulations.

As an example, we investigate the scenario of pumping the TFLN chip (with waveguide dimensions as in our experiments) with Fourier-limited Gaussian pulses with a FWHM intensity duration of 500 fs and 1 nJ energy (10 times higher than in our experiments). Our findings reveal that using such a pulse length still contributes to optical rectification up to 500 GHz. However, shortening the pulse (increasing the peak power), would result in strong

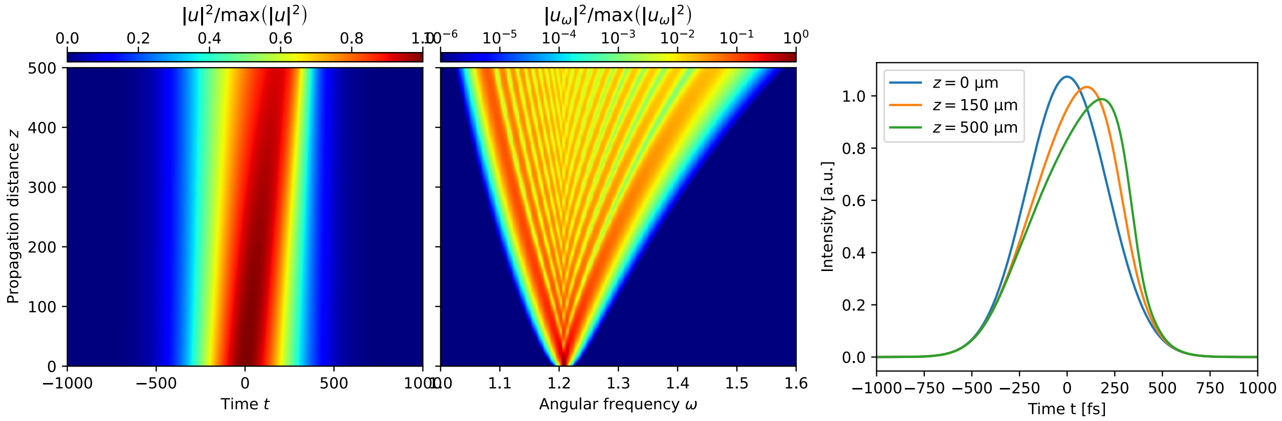

Supplementary Fig. 8: Simulation of a 500 fs-long Gaussian pulse with a pulse energy of 100 nJ in a 0.5 mm long TFLN waveguide performed with the python package fmas [5]. Left: Evolution of the temporal pulse-shape. Middle: Signal evolution in the spectral domain. Right: Pulse shape after the propagation length of a typical antenna gap length (150 µm) and a device of several antennas (0.5 mm) compared to the initial pulse shape.

nonlinear effects. We do not find any significant change in the temporal pulse shape up to distances of 4 mm for our given waveguide geometry. Hence, we predict the terahertz generation to remain unaffected for this length (Supplementary Fig. 7). In the frequency domain we can see the spectral broadening due to nonlinear effects (Supplementary Fig. 7). Further, we find that pulses of 100 nJ (1000 times higher than in our experiment) propagate without significant pulse broadening over a length of 500 µm, even if strong spectral broadening occurs (Supplementary Fig. 8). From these simulations, we conclude that pulse energies in the range of $\sim$ 100 nJ will be the upper power limit. If successfully guiding a 500 fs pulse into one of our antenna gap, the amplitude of such a single-antenna emission is expected to increase by 3 orders of magnitude to $1\,\mathrm{kV\,m^{-1}}$ and the pulse energy of the THz signal can reach $\sim$ 10 fJ.

Supplementary Note 4 (Simulation of antenna structure)

The simulations of the THz field emitted by different antenna structures in this work were all computed by the commercial simulator software of CST Microwave studio. The sample layer thicknesses and antenna dimensions were chosen similar to the values listed in Supplementary Table 1, except of the substrate thickness. To suppress the effect of a Fabry-Perot-etalon in the investigated frequency range, the substrate thickness was reduced to 30 µm. Aiming for a shorter computation time, the simulated area in the sample plane was adapted to the antenna size (Supplementary Fig. 9 a) and the simulated frequency range is chosen according to the particular expected resonance frequency. A discrete port placed in the center of antenna gap excites the antenna. For a broadband investigation the scenario is simulated using the time-domain solver. To determine the strength of the emitted radiation, a field probe is placed centered below the device in 500 µm distance. The determined electric field at the probe E_{probe} (solid lines in Supplementary Fig. 9 b) is normalized with the field calculated for a similar port and probe but without any antenna structure E_{ref} (grey dotted line in Supplementary Fig. 9 b). The obtained filter function $R(\Omega) = \frac{E_{\mathrm{probe}}}{E_{\mathrm{ref}}}$ shows the spectral radiation properties of the particular antenna design (Supplementary Fig. 9 c), whereby the frequency of maximum $R(\omega)$ identifies its peak frequency. Since the length of the generation area strongly affects the emitted intensity, it needs to be taken into account for investigating the emission strength of different antenna designs. Thus the normalized antenna emission $R(\Omega)$ is multiplied by the spectral field distribution for optical rectification $E_{\mathrm{OR}}(\Omega)$

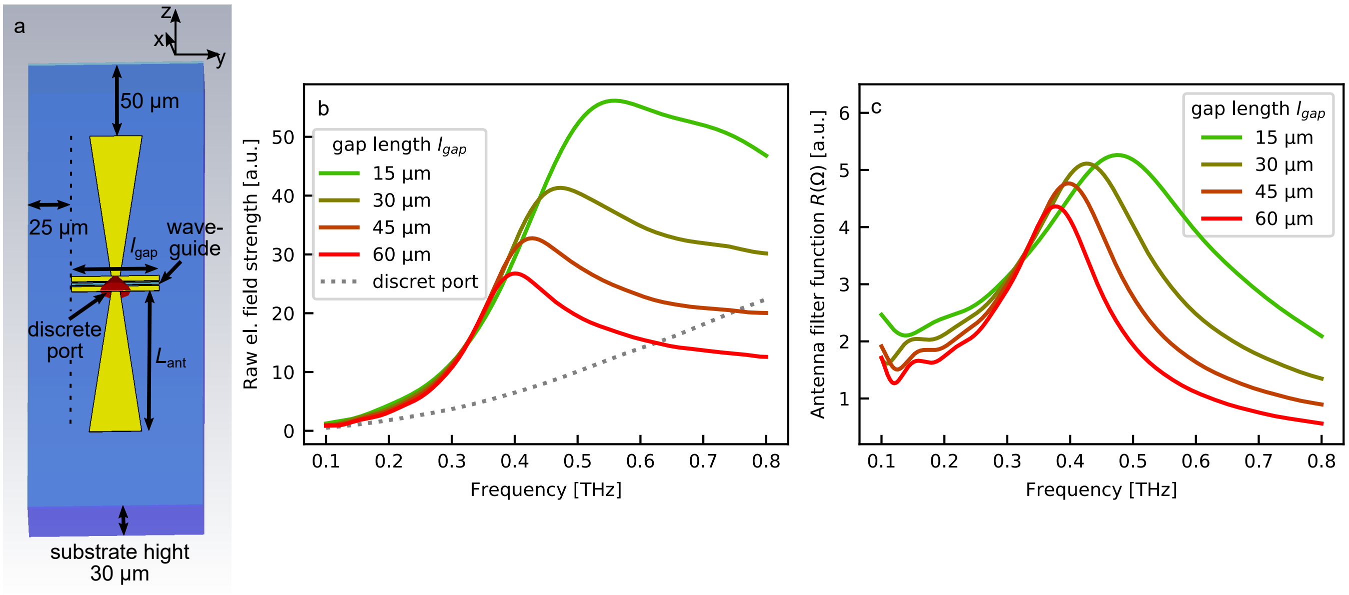

Supplementary Fig. 9: Schematic of structure used for simulations. a: THz antenna on thin-film lithium niobate excited by a discrete port. **b**: Simulated electrical field emitted by antennas of different gap length l_{gap} (solid colored lines) in comparison to the emission of a bare discrete port without any antenna structure (dashed grey line). **c**: Filter function $R(\Omega)$ of same antenna simulations as in b.

including the effect of different effective generation lengths l_{eff}:

$$I_{\text{sim}}(\Omega) \sim R^2(\Omega) \cdot I_0^2(\Omega) \cdot \Omega^2 \cdot l_{\text{eff}}^2(\Omega). \tag{16}$$

The peak value of $I_{\text{sim}}(\nu)$ gives a measure for the emitted intensity depending on the antenna parameters or the properties of the pump pulse.

A Antenna size study

First, we present the simulation of the emission of antennas with different antenna sizes similar to the experimental study (Fig. 2 c of the main text). For comparability of the determined emission strength the simulated volume and the frequency range is kept identical being sufficient for all simulated antenna dimensions. The peaks of the simulated response $R(\Omega)$ drift towards higher frequencies for decreasing antenna size, and a broadening and a weakening of the response function is observable (Supplementary Fig. 10 a). Especially for larger antennas ($L_{\text{ant}} \geq 50\,\mu\text{m}$) the frequency of the maximal response function matches the experimentally determined values (Supplementary Fig. 10 b) underlying the emission frequency being controlled by the antenna design. In line with the experimental study, we adapt the antenna gap length $l_{\text{gap}} = \frac{L_{\text{ant}}}{2}$ to the antenna length L_{ant} ensuring a generation length below the coherence length. According to equation (8), the field generated by optical rectification weakens with decreasing generation length. Nevertheless the decreasing gap length of smaller antennas is compensated by the increasing resonance frequency, which can be approximated to follow the relation $\Omega_{\text{res}} \sim \frac{1}{L_{\text{ant}}} = \frac{1}{2l_{\text{gap}}}$. Consequently the antenna response function $R(\Omega)$ and the Fourier transformation of the pump's temporal intensity envelope $I(\Omega)$ determine the observed signal strength emitted by different antennas of this particular design. We estimate the generating intensity envelope $I_0(t)$ by Fourier transforming the spectrum measured after propagating through the chip (blue line in Supplementary Fig. 10 c). As discussed in Supplementary Note 3 A, the spectral phase is assumed to be exclusively caused by the dispersion of the single-mode fiber (1.1 m SMF 28 from Thorlabs, setup details in Supplementary Note 5 A)

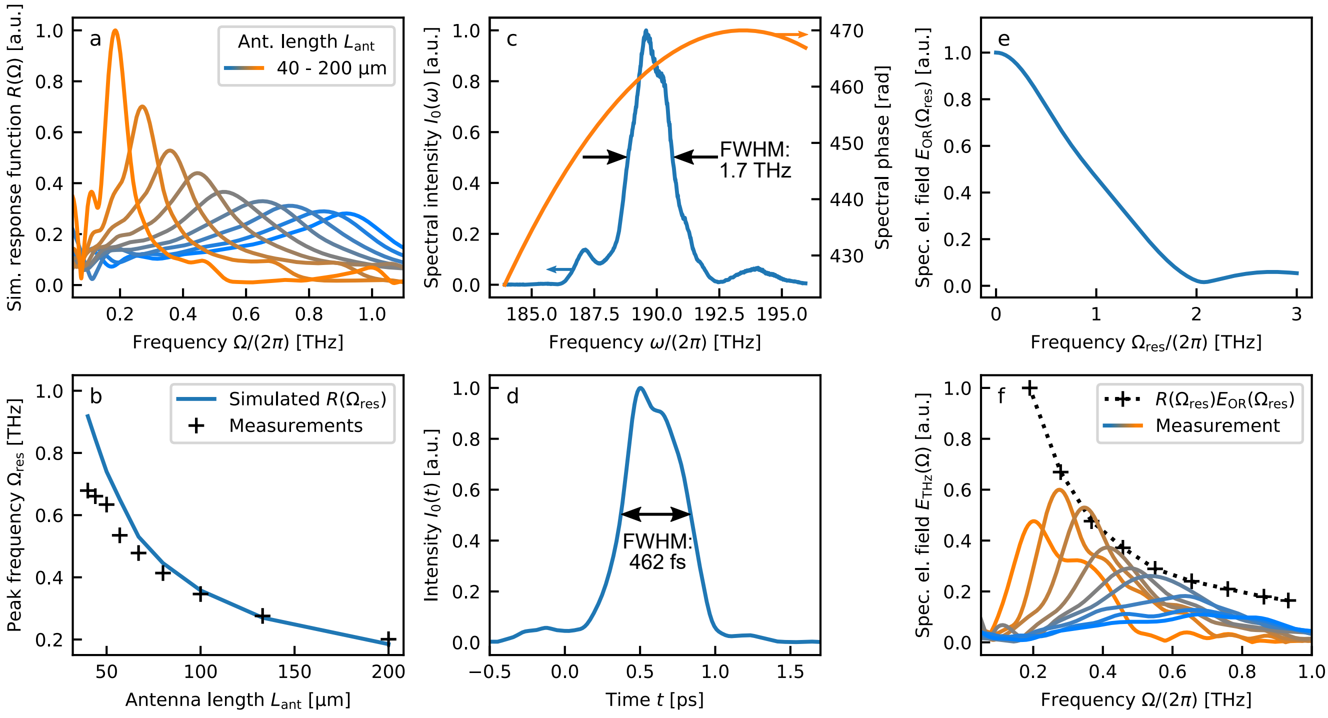

Supplementary Fig. 10: Emission frequency constrains. **a**: Antenna response function $R(\Omega)$ simulated with CST Microwave Studio for bow-tie arm lengths L_{ant} between 40 µm and 200 µm similar to the experimentally investigated antenna designs (Fig. 2 c of the main text). **b**: Comparison of the peak frequency Ω_{res} determined from the measured antenna emission (black crosses) and the antenna response function $R(\Omega)$ (solid blue line) depending on its antenna arm length L_{ant}. **c**: Spectral intensity $I_0(\omega)$ of the pump signal (blue line, left vertical axis) and spectral phase caused by dispersion inside the fiber (orange line, right vertical axis). The full width at half maximum intensity of the spectrum measures 1.7 THz. **d**: Simulated pump intensity in time-domain $I_0(t)$ with a full width half at half maximum intensity pulse duration of 462 fs. **e**: THz field E_{OR} generated in the antenna gap by optical rectification of the pump pulse shown in (d), evaluated at the resonance frequency Ω_{res} when the gap length is linked to the antenna size antenna via $l_{\text{gap}} = \frac{L_{\text{ant}}}{2}$. **f**: Measured spectral electrical field emitted by antennas of arm lengths between 40 µm and 200 µm (solid lines) in comparison with the expected signal strength at the resonance frequency Ω_{res} of the different antenna designs (black crosses connected by dashed line).

guiding the pulses onto the TFLN chip (orange line in Supplementary Fig. 10 c) and the dispersion inside the on-chip waveguide. The influence of nonlinear effects inside the fiber or the waveguide are neglected due to the comparable low power of 31 mW in this particular study leading to a pump signal of 462 fs pulse duration (Supplementary Fig. 10 d). Based on these assumptions the electric field exciting the antenna $E_{\text{OR}}(\Omega_{\text{res}})$ is maximal in the limit of 0 THz and decreases in strength extending up to almost 2 THz (Supplementary Fig. 10 e). In combination with the decreasing emission efficiency observed in the antenna simulation the trend of the expected signal strength at the particular resonance frequency explains the experimentally observed signal behavior (Supplementary Fig. 10 f). Only in case of small antenna sizes ($L_{\text{ant}} \leq 50$ µm) the measured field strength deviates from the predicted curve. Due to the larger focal spot size, low frequencies are less sensitive to slight misalignment of the sample with respect to the collecting parabolic mirror. Additionally, also the overlap between THz and probe becomes more critical to alignment mistakes for higher frequencies and therefore lower spot sizes. Both effects lead to a less efficient detection of higher frequencies explaining the shift to lower frequencies and weaker signals emitted by the smaller antennas ($L_{\text{ant}} \leq 50$ µm).

B Gap length study

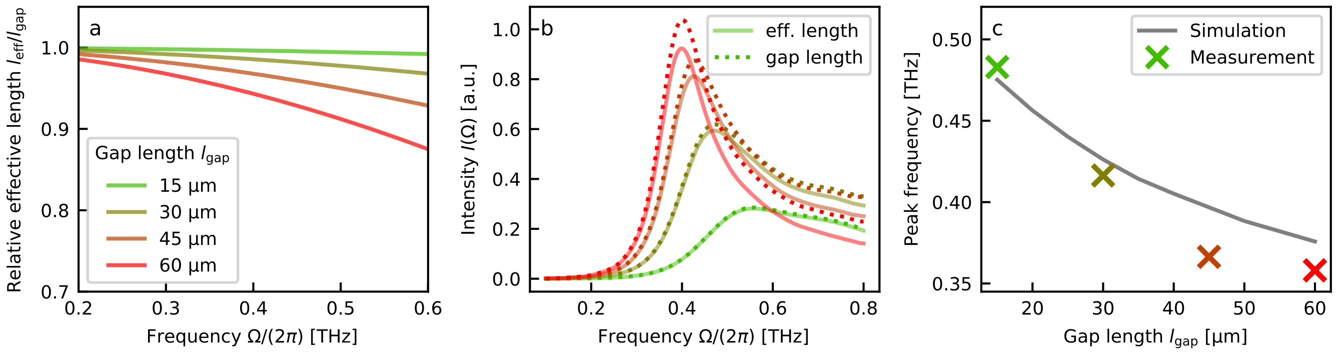

Supplementary Fig. 11: Impact of gap length on emitted THz field a: Effective generation length normalized to the gap length for the different gap dimensions investigated in Fig. 2 d-f of the main text. **b**: Simulated spectral intensity emitted from devices of different gap length corresponding to a. The solid line shows the simulation taking into account the frequency dependent effective generation $l_{\mathrm{eff}}(\Omega)$ described in equation (3), while the dashed line neglects the influence of phase matching assuming $l_{\mathrm{eff}} = l_{\mathrm{gap}}$. **c**: Simulated peak frequency according to the the product of the antenna resonance and the effective generation length $R(\Omega) \cdot l_{\mathrm{eff}}(\Omega)$ compared to the measured values (Fig. 2 d of main text).

In the following we investigate the influence of the gap length on the emitted THz field, discussed in Fig. 2 d-e of the main text. The frequency dependent effective generation length l_{eff} described in equation 7 can influence the spectral properties of the emitted THz signal. Therefore the ratio between l_{eff} and the true gap length l_{gap} is plotted in Supplementary Fig. 11 a for the experimentally investigated devices. Within the relevant frequency range between 200 GHz and 600 GHz even for the longest gap of 60 µm the effective generation length decreases by less than 15 %, so that the shift of the measured peak from 483 GHz to 358 GHz cannot be explained only by phase matching. In Supplementary Fig. 11 b the simulated response function $R(\Omega)$ is once multiplied with the effective generation length $l_{\mathrm{eff}}(\Omega)$ (solid line) and in comparison to that with the full gap length l_{gap} (dotted line). The main difference is a increase intensity assuming the full generation length but the spectral properties are almost identical. The comparison of the measured and simulated peak frequencies shows an accordance explaining the spectral shift (Supplementary Fig. 11 c). In conclusion for the gap lengths investigated in this work, which are below the coherence of $l_{\mathrm{coh}} = 64\,\mu\mathrm{m}$ calculated for a frequency of 500 GHz, the phase matching within the antenna gap has a minor influence on the emitted spectrum.

C Control of THz polarization

Since the highest nonlinear coefficient of lithium niobate - and therefore the most efficient THz generation - occurs along the [001]-axis, within our experimental study, all the antenna structure is always oriented to emit THz fields polarized along this axis. To expand our platform by a new degree of freedom in THz control, we investigate modified antenna designs keeping the orientation of the waveguide inside the antenna gap along the y-axis of the lithium-niobate while we control the polarization of the emitted wave through the design of the antenna alone. Supplementary Fig. 12 shows the simulation results for two new antenna geometries on the same material platform as in the manuscript. By departing from the standard bow-tie geometry, we show that we can control the polarization state of the emitted THz wave. In Supplementary Fig. 12 a an antenna with bowtie-like arms is placed parallel to the y-axis of the crystal. We choose an antenna arm length of $L_{\mathrm{ant}} = 90\,\mu\mathrm{m}$ and a gap length of $l_{\mathrm{gap}} = 45\,\mu\mathrm{m}$ keeping the parameters similar

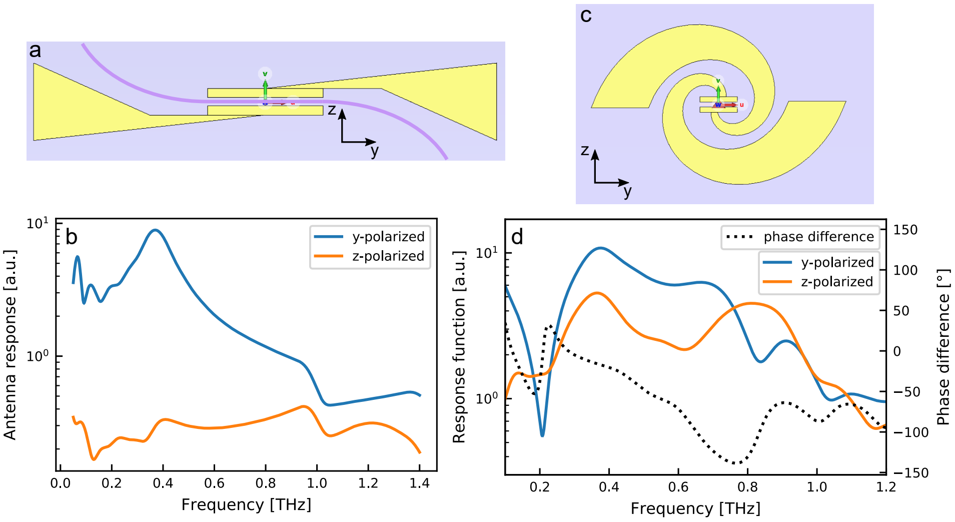

Supplementary Fig. 12: **a**: Antenna structure for THz emission perpendicular to the excitation direction. The dimensions are similar to the ordinary bowtie antenna (reported in Supplementary Note 4) using an antenna arm length of $L_{\text{ant}} = 90\,\mu\text{m}$ and a gap length of $l_{\text{gap}} = 45\,\mu\text{m}$. **b**: Antenna response of the structure shown in (a) along the y- and z-axis simulated with CST Microwave studio plotted in logarithmic scale. **c**: Spiral antenna structure for elliptically polarized THz emission. A logarithmic spiral with an inner radius $r = 10\,\mu\text{m}$, phase-shift of $\delta = 90°$ and progression of $\alpha = 0.35$ is placed around the antenna gap formed by two gold bars of length $l_{\text{gap}} = 25\,\mu\text{m}$ defining the generation area. **d**: Antenna response simulated with CST Microwave studio for the log-spiral antenna shown in c along y- and z-direction and the phase difference between the two different polarization directions.

to the original bow-tie antenna designs. Even when keeping the THz excitation along the z-axis, the emitted signal is mainly polarized along the y-axis (Supplementary Fig. 12 b). In other words, the antenna rotates the polarisation of the outcoupled THz field. Our simulations reveal that, in this case, the field ratio between the z-polarization and the y-polarization is below 4 % at the resonance frequency of 370 GHz, corresponding to an intensity extinction ratio of -28 dB. This demonstrates that y-polarised terahertz emission is achieved by such an antenna structure.

Following the same principle, log-spiral antennas (Fig 12 c) can be employed to generate elliptically polarized THz signals [8]. The log-spiral with an inner radius $r = 10\,\mu\text{m}$, phase-shift of $\delta = 90°$ and progression of $\alpha = 0.35$ is placed around the antenna gap formed by two gold bars of length $l_{\text{gap}} = 25\,\mu\text{m}$ defining the generation area. We find that such antenna design can generate elliptically polarized light (Fig 12 d).

Supplementary Note 5 (Supporting Measurements)

A Time-resolved THz electric field measurement

The principle of dual-wavelength time-domain spectroscopy is utilized in the present work to investigate the signal generated by on-chip optical rectification (Supplementary Fig. 13). The Er:fiber-based laser system provided by MenloSystem emits a pulsed signal at 1560 nm and its second harmonic at 780 nm. Since both signals origin from the same laser cavity, the pulses are perfectly synchronized at a repetition rate of 60 MHz. The pump signal centered around 1560 nm is coupled into 1.1 m of single mode fiber ending in a cleaved fiber tip. Due to the high peak power of initially 70 fs short pulses, the spectra after 1 m of SMF is changed by nonlinear effects inside the fiber depending on the pulse energy (Supplementary Fig. 13 panel I). To optimize the fiber tip (FT) position a second cleaved fiber collects the light transmitted through the chip at the output grating coupler. By the use of two 3D-piezo-stages provided by SmarAct with nm-precision, both fiber tips are placed into the position of maximum transmitted power.

The coupling efficiency of the grating couplers is characterized in Supplementary Note 5 C. A pair of identical parabolic mirrors with a numerical aperture of 0.45 collimates and focuses the emitted radiation into a 3 mm ⟨110⟩ zinc telluride detection crystal (DTX), where it is overlapped with the probe signal centered at 780 nm.

The nonlinear interaction between the THz and near-infrared signal inside the detection crystal maps the electric THz field onto the polarization state of the probe beam. Measuring the polarization change in the probe signal accesses the information of the electric field strength of the THz signal within time-overlap of the probe signal. By stepwise delaying the probe pulses with respect to the THz signal, the electric field of the generated THz is sampled in time-domain, which is also known as electro-optic sampling. Thereby the time-resolution is limited by the pulse-length of the probe signal. The auto-correlation determines a pulse length of 180 fs of the probe pulses (Supplementary Fig. 13 panel II). Consequently, the achievable frequency-limit of about 10 THz is above the frequency range of the investigated signals (< 1 THz). In our case the THz field is polarized along the [-110] crystallographic axis and the probe signal along [001], so that the THz field strength is given by [9]:

$$E_{\mathrm{THz}}(t) = \frac{c \cdot \Delta I}{\omega_0 \cdot n^3 \cdot r_{41} \cdot l_{\mathrm{ZnTe}} \cdot I_{\mathrm{probe}}}, \tag{17}$$

where ΔI denotes the intensity difference at the two diodes of the balanced detector (BD), ω_0 the center angular frequency of the probe pulses, $n = 2.87$ the refractive index of zinc telluride in the near-infrared, $r_{41} = 3.9\,\mathrm{pm/V}$ it's second order nonlinear coefficient, $l_{\mathrm{ZeTe}} = 3\,\mathrm{mm}$ the crystal length and I_{probe} the intensity of the probe inside the detection crystal. The Fourier-transformation of the measured time-trace delivers the measured spectral field $E_{\mathrm{OR}}(\Omega)$, whereby Ω always denotes the angular frequency.

B THz polarization

In the configuration of the probe pulse polarized along the [001]-axis of the detection crystal, electro-optic sampling is sensitive to electric THz fields along the [-110]-axis, whereby the chip is in all cases oriented to have the bow-tie antenna along this direction. To check for THz emission along the perpendicular polarisation, the crystal is turned by 90°, keeping the original probe polarization, which is now along the [-110]-direction of zinc telluride. In this configuration the crystal is still exclusively sensitive to THz fields along the [-110]-axis, which is now perpendicular to the bow-tie antenna. Since no signal is observable for the turned configuration, as can be seen in to comparison to a regular measurement (Supplementary Fig. 14), the antenna emits linear polarized THz radiation, as expected.

C Coupling of optical signals

The near-infrared power pumping the THz on-chip are determined for different configurations, since the measurements presented in the main text are taken for different incoming powers and samples. Inside the optical fiber guiding the pump-pulse to the on-chip grating couplers, the high peak power leads to nonlinear self-phase modulation. Consequently the input spectrum before coupled to the chip, as can be seen in Supplementary Fig. 15 a for the two input power configurations of 64 mW and 31 mW in the present work. Since the coupling efficiency of the gratings is frequency dependent, a spectral change leads also to a different coupling efficiency. Additionally chips origin from two separate fabrication processes are investigated, whereby a significant difference in coupling efficiency is observed (Supplementary Fig. 15 b and c). For the study of different antenna sizes (Fig. 2 c of the main text) done with chip 2, the input power of the pump signal was reduced to 31 mW leading to a higher spectral width behind the chip. This enabled us to reach the higher frequencies of small antenna's, since the bandwidth of optical rectification is influenced

by the pulse length and the bandwidth of the pump signal. To determine the power on chip, we first calculate the frequency-dependent ratio between outgoing and incoming spectral density:

$$\mathrm{d}P_{\mathrm{out}}(\lambda) = C(\lambda) \cdot \mathrm{d}P_{\mathrm{in}}(\lambda). \tag{18}$$

Assuming a equal influence of the input and output grating coupler on the spectral density, the power density on chip is directly calculated as:

$$\mathrm{d}P_{\mathrm{chip}}(\lambda) = \sqrt{C(\lambda)} \cdot \mathrm{d}P_{\mathrm{in}}(\lambda). \tag{19}$$

By integration the power on-chip, the corresponding pulse energy is determined. In the case of the first chip, a pulse energy 98 pJ is determined, while for the second chip in the case of 70 mW (31 mW) a pulse energy of 40 pJ (16 pJ) is coupled into the waveguide.

D Efficiency of THz generation

To determine the generated THz pulse energy from the measured electric field in time-domain, we first calculate the root-mean square of the measured electrical field E_{rms} within a time-interval Δt covering the full THz pulse. The intensity averaged over the time-interval Δt is given by:

$$I_{\mathrm{pulse}} = \frac{c \cdot n \cdot \varepsilon_0}{2} \cdot |E_{\mathrm{amp}}|^2 = c \cdot n \cdot \varepsilon_0 \cdot |E_{\mathrm{rms}}|^2. \tag{20}$$

From the spatial measurements presented in Supplementary Note 5 F we find a Gaussian width of the THz focus of $w_0 = 1.7\,\mathrm{mm}$, so that the pulse energy is calculated by:

$$E_{\mathrm{pulse}} = \frac{\pi}{2} \cdot f w_0^2 \cdot \Delta t \cdot I_{\mathrm{pulse}}. \tag{21}$$

We achieve THz pulse energies in the order of 10^{-20} J leading to an efficiency of $\sim 10^{-10}$, which is defined as the ratio between the generated THz pulse energy ($\sim 10^{-20}$ J) and the pump pulse energy on chip (98 pJ).

E Pump power dependency

According to equation (8) the spectral electric field $E_{\mathrm{OR}}(\Omega)$ generated via optical rectification is proportional to the input optical intensity $I_0(\Omega)$. In this section we investigate the THz signal emitted by a 3-antenna array emitting radiation mainly at 205 GHz for changing power of the incoming pump signal between 4.9 mW and 58.7 mW before coupled into the chip. For this device the nonlinear effects of the pump signal inside the coupling fiber does not influence the generated signal significantly, since its emission frequency is low enough to not be affected by slight changes of the pump pulse shape (Supplementary Fig. 16 a). The measured field distributions, which are plotted in Supplementary Fig. 16 a, confirms a decrease in the THz signal while maintaining the spectral shape. Moreover, the evaluation of the peak electric field of the main peak around 205 GHz (blue curve) and of the side peak around 425 GHz (orange curve in Supplementary Fig. 16 b, left axis) confirms the linear relationship to the incoming power. The THz pulse energy (green line in Supplementary Fig. 16 b) shows the quadratic dependency typical for second order nonlinear process, as expected.

F Spatial map of cascaded antenna emission

To investigate the spatial properties of a three antenna array , the sample is moved within the focal plane of the collecting parabolic mirror. Shifting the device perpendicular to the waveguide direction (Supplementary Fig. 17 a), the signal strength in time domain (Supplementary Fig. 17 b) as well as in the frequency domain decrease with the distance from the center antenna while the spectral shape remains similar (Supplementary Fig. 17 c). By fitting the peak spectral intensity with a Gaussian function, the beam waist within the focus is determined to measure 1.69 mm (Supplementary Fig. 17 d). For a moving direction along the optical waveguide (Supplementary Fig. 17 e), the signal envelope changes in time-domain (Supplementary Fig. 17 f). Since the distance between the different arrays measuring 0.58 mm is in the order of the focal spot size, the peak associated with the antenna closest to the focal point in the particular configuration appears to be the strongest. Additionally, the difference in THz path length discussed in Supplementary Note 2 C becomes observable even though the large aperture mirror collects signals emitted under different emission angles. Moving the focus against the propagation direction (negative direction, blue lines), the THz signal triggered first, travels the shortest propagation path leading to a slight shift of the spectrum towards lower frequencies (Supplementary Fig. 17 g). In contrast, for the opposite direction (orange lines), a shift of the spectrum towards higher frequencies is observed, since the THz phase shift counteracts against the time-delay caused by the propagation path of the optical pump signal. Due to the extension of the devices itself of ~ 1.2 mm, the measured spot sizes along the waveguide direction increases to 1.95 mm, whereby the Gaussian shape is still observable (Supplementary Fig. 17 h).

Supplementary Note 6 (Comparison with other terahertz generation schemes)

In this work, we depart from the commonly employed approach to generate terahertz transients in bulk nonlinear crystals and instead demonstrate on-chip generation that explores many of the advantages of integrated photonic circuits. Nevertheless, various prior works have attempted to generate terahertz transients with various characteristics. Since most of the works so far however are performed in bulk nonlinear crystals (among which lithium niobate) or by shaping the terahertz waveform after its generation by pulse shaping elements positioned after the emitter, our approach here using integrated circuits is an entirely different approach.

We provide in the table below a comparative analysis with some of the works that have succeeded in the past to custom-tailor the emission of terahertz transients. Approaches based on quasi-phase matching as e.g. periodically poled LN generate narrowband multi-cycle radiation, where the emission frequency is controlled by the domain period [10, 11, 12, 13, 14]. In fanned-out PPLN, the spatially varying domain period allows to control the frequencies contributing to the THz signal [15]. Designing PPLN the signal strength emitted from each individual domain is directly linked to the domain thickness. In contrast, in our case, the distance of the antennas can be varied independently from the gap length determining the signal strength giving a further degree of freedom in the waveform generation. TFLN offers the ability of poling the LN as well allowing more efficient devices by reducing the required antenna distance. While in PPLN the number of periods is limited by absorption of THz signal, the perpendicular antenna emission circumvents THz propagation though the LN. Due to the low optical losses in TFLN waveguides, the number of antennas can be further increased, mainly limited by the desired chip size. Poling of LN allow the modification of the crystallographic structure only along the [001] axis, so that the THz polarization is fixed to one direction within a PPLN crystal. The overlap of THz pulses via photo-injected reflection offers a almost similar degree of flexibility [16], but also in this approach the THz field is determined by the parallel-plate waveguide preventing any

polarization modification. THz shaping based on diffractive surfaces is another promising approach offering besides the spectral and temporal tuning the control over the polarization properties of the THz radiation. However, all the above mentioned approaches rely on the overlap of many single THz pulses determining spectral and temporal properties. Inherently, these approaches are not suited for the generation and control of few- or single-cycle THz pulses with broadband spectra. Using single or parallel antenna emitters, we have the additional possibility to few-cycle pulses maintaining the control of the center frequency by the antenna design. Using GaP instead of LN, the crystallographic structure allows the generation of single-cycle THz field along two perpendicular axis depending on the pump signal's polarization state. Consequently shaping the polarization of the incoming pulses by the use of an optical pulse shaper gives the full control of the THz polarization [17]. This approach might be combined with quasi-phase-matched GaP generating many-cycle waveforms combined with polarization control. Nevertheless we do not see the opportunity to also control the spatial pattern of the THz radiation, as the distribution of several antennas within the 2D-chip plane of TFLN can offer. Within a nonlinear meta-surface the variation of the antenna orientation is utilized to create custom-tailored spatial THz field pattern, but this approach is restricted to the generation of single-cycle pulses missing the possibility of arbitrary waveform generation [18].
Consequently, using integrated photonic circuits provides unique flexibility of tailoring THz radiation within a single technology and this approach shows the potential to be further developed into a fully integrated design.

	method	broad	narrow	polarization	spatial	at source
**Our work** Integrated circuits in TFLN	On-chip circuits	✓	✓	✓(sim.)	✓	✓
Quasi-phasematched crystals [10, 11, 12, 13]	bulk	✗	✓	✗	✗	✓
Fanned-out PPLN [15]	bulk	✗	✓	✗	✗	✓
Polarization shaping [17]	bulk	✓	(✓)	✓	✗	✓
Photo-injected reflection [16]	p.s.a.e.	✗	✓	✗	✗	✗
Nonlinear Metasurfaces [18]	free-space	✓	✗	(✓)	✓	✓
Diffractive surfaces [19]	p.s.a.e.	✗	✓	✓	✓	✗

Supplementary Table 2: Comparison of the flexibility in the THz properties offered by integrated circuits in TFLN to common THz generation approaches. p.s.a.e = pulse shaping after the emission e.g. through spatial light modulators placed downstream from the emitter, sim = simulations only.

As a result of this comparison, we now underline the clear unique advantages that our platform provides. While other works covers certain degrees of freedom in tailoring the THz radiation, our work can control all properties of the THz wave using a single platform (see table 2).

1. **the on-chip waveguides** provide compactness and compatibility with fiber-based source and detector tech-

nologies. This is critical since experiments using conventional Ti:Sapphire lasers suffer from huge pulse broadening due to large GVD of standard optical fibers. Also, by using available telecommunication infrastructure experiments can be done without the challenge of free-space propagation of the pump pulses,

2. **the design of the individual antenna and the antenna distribution on-chip** provides the possibility to custom-tailor the radiation at the source - rather than through a spectral shaping element placed after a broadband emitter (which is the case for spatial light modulators [20]), thereby minimizing the effective loss,

3. **the low propagation loss characteristic for our fabrication process** allows for complex photonic architectures, where several antennas may be placed downstream rather than using discrete components with considerable insertion losses,

4. **antenna structures can also be used to pole the lithium niobate film**,

5. **the thermal and mechanical stability** of chip-based devices. For example, the operation point of integrated interferometers has better stability compared to hand-made free-space ones,

6. **the simultaneous control over several degrees of freedom** by chip-scale design alone (i.e. in our case all emitters are pumped under the same conditions, just the chip design is changed),

7. **the control of the pump-pulse propagation both temporally (compression or broadening) and spectrally** using waveguide geometric dispersion [21, 6]. For example, by changing the dimensions of the waveguide, one can launch desired pulses with proper characteristics to the antenna where the THz generation occurs.

8. outlook on combining our demonstration with active chip-scale components that are intrinsically fast (e.g. GHz-speed intensity modulators to time the pump pulses, multi-port splitters to distribute pump pulses on-chip, or integrated laser sources compatible with TFLN towards fully-integrated THz sources [22]),

9. outlook to combine the emitters with perfectly matched detectors on the same chip.

Supplementary Note 7 (Increase of generated THz power)

While we achieve THz fields in the range of only $1\,\mathrm{V\,m^{-1}}$ within the current study, various optimizations of the demonstrated on-chip THz source may allow the generation of stronger THz signals in the future.

First of all, one could imagine using the available pump power on-chip more efficiently to avoid most of the pump remaining undepleted. One can use several subsequent antennas to improve efficiency. Additionally, periodic poling can be employed to increase the effective number of antennas by a factor of 2 by flipping the crystal domains. Also, the number of parallel channels may be increased as well, for example by placing 20×20 or more antennas on chip. In this case, the temporal pulse spreading can be managed by tailoring the waveguide dispersion as in refs.[21, 6].

In our current investigation the pulse energy of the pump signal measures only 100 pJ. In Supplementary Note 3 B we show that for an increase of the of the pump energy to 100 nJ self-phase modulation of the pump signal within the generation area will affect the THz generation. Consequently the emitted terahertz field amplitude can potentially be increased by a factor of 1000. By implementing 20 antennas in parallel, we project that the field amplitudes on the order of $20\,\mathrm{kV\,m^{-1}}$ should be achievable for this system.

Finally, the THz energy reported in the present work is the as-measured value ignoring any loss in the system. For instance, the THz energy reported is what is transmitted through ≈30 cm of unpurged path length, collected

with parabolic mirrors and focused onto the detection crystal without any correction. By plugging in the numbers, we find that due to the high refractive index of the silicon substrate in the THz range ($n_{\mathrm{SI}} = 3.425$ [23]), the angular width (110° according to the farfield in CST simulations, NA = 0.82) exceeds the parabolic mirror (NA = 0.5). Consequently, the measured THz pulse energy is lower than the full signal emitted by the device by an estimated factor of 2.7. A silicon lens can be attached directly into the substrate to improve the collection for the directed farfield, similar to commercial photoconductive emitters. [24, 25].

In conclusion, the above improvements are expected deliver terahertz field amplitudes of about $20\,\mathrm{kV\,m^{-1}}$, which is on-par with other electro-optic emitters used e.g. in time-domain spectroscopy, sensing.

Supplementary References

[1] C. Rønne, L. Thrane, P.-O. Åstrand, A. Wallqvist, K. V. Mikkelsen, and S. R. Keiding. "Investigation of the temperature dependence of dielectric relaxation in liquid water by THz reflection spectroscopy and molecular dynamics simulation." The Journal of Chemical Physics, **107**(14):5319–5331 (1997).

[2] A. Schneider, M. Neis, M. Stillhart, B. Ruiz, R. U. A. Khan, and P. Günter. "Generation of terahertz pulses through optical rectification in organic DAST crystals: theory and experiment." Journal of the Optical Society of America B, **23**(9):1822 (2006).

[3] M. G. Raymer and I. A. Walmsley. "Temporal modes in quantum optics: then and now." Physica Scripta, **95**(6) (2020).

[4] B. Brecht, D. V. Reddy, C. Silberhorn, and M. G. Raymer. "Photon Temporal Modes: A Complete Framework for Quantum Information Science." Physical Review X, **5**(4) (2015).

[5] O. Melchert and A. Demircan. "A Python Package for Ultrashort Optical Pulse Propagation in Terms of Forward Models for the Analytic Signal." Computer Physics Communications, **273**:108257 (2022).

[6] D. Zhu, L. Shao, M. Yu, R. Cheng, B. Desiatov, C. J. Xin, Y. Hu, J. Holzgrafe, S. Ghosh, A. Shams-Ansari, E. Puma, N. Sinclair, C. Reimer, M. Zhang, and M. Lončar. "Integrated photonics on thin-film lithium niobate." Adv. Opt. Photon., **13**(2):242–352 (2021).

[7] M. Bache and R. Schiek. "Review of Measurements of Kerr Nonlinearities in Lithium Niobate: The Role of the Delayed Raman Response." (2012). ArXiv preprint arXiv:1211.1721.

[8] W. Miao, Y. Delorme, F. Dauplay, G. Beaudin, Q. J. Yao, and S. C. Shi. "Simulation of an Integrated Log-Spiral Antenna at Terahertz." In "2008 8th International Symposium on Antennas, Propagation and EM Theory," pages 58–61. IEEE, Kunming, China (2008).

[9] P. C. M. Planken, H.-K. Nienhuys, H. J. Bakker, and T. Wenckebach. "Measurement and calculation of the orientation dependence of terahertz pulse detection in ZnTe." Journal of the Optical Society of America B, **18**(3):313 (2001).

[10] Y.-S. Lee, T. Meade, V. Perlin, H. Winful, T. B. Norris, and A. Galvanauskas. "Generation of narrow-band terahertz radiation via optical rectification of femtosecond pulses in periodically poled lithium niobate." Applied Physics Letters, **76**(18):2505–2507 (2000).

[11] Y. S. Lee, T. Meade, T. B. Norris, and A. Galvanauskas. "Tunable Narrow-Band Terahertz Generation from Periodically Poled Lithium Niobate." Applied Physics Letters, **78**(23):3583–3585 (2001).

[12] Y.-S. Lee and T. B. Norris. "Terahertz Pulse Shaping and Optimal Waveform Generation in Poled Ferroelectric Crystals." Journal of the Optical Society of America B, **19**(11):2791 (2002).

[13] T. Buma and T. B. Norris. "Coded Excitation of Broadband Terahertz Using Optical Rectification in Poled Lithium Niobate." Applied Physics Letters, **87**(25):251105 (2005).

[14] G. Imeshev, M. E. Fermann, K. L. Vodopyanov, M. M. Fejer, X. Yu, J. S. Harris, D. Bliss, and C. Lynch. "High-Power Source of THz Radiation Based on Orientation-Patterned GaAs Pumped by a Fiber Laser." Optics Express, **14**(10):4439 (2006).

[15] J. R. Danielson, N. Amer, and Y.-S. Lee. “Generation of Arbitrary Terahertz Wave Forms in Fanned-out Periodically Poled Lithium Niobate.” Applied Physics Letters, **89**(21):211118 (2006).

[16] L. Gingras and D. G. Cooke. “Direct Temporal Shaping of Terahertz Light Pulses.” Optica, **4**(11):1416 (2017).

[17] M. Sato, T. Higuchi, N. Kanda, K. Konishi, K. Yoshioka, T. Suzuki, K. Misawa, and M. Kuwata-Gonokami. “Terahertz Polarization Pulse Shaping with Arbitrary Field Control.” Nature Photonics, **7**(9):724–731 (2013).

[18] S. Keren-Zur, M. Tal, S. Fleischer, D. M. Mittleman, and T. Ellenbogen. “Generation of Spatiotemporally Tailored Terahertz Wavepackets by Nonlinear Metasurfaces.” Nature Communications, **10**(1):1778 (2019).

[19] M. Veli, D. Mengu, N. T. Yardimci, Y. Luo, J. Li, Y. Rivenson, M. Jarrahi, and A. Ozcan. “Terahertz Pulse Shaping Using Diffractive Surfaces.” Nature Communications, **12**(1):37 (2021).

[20] W. L. Chan, H.-T. Chen, A. J. Taylor, I. Brener, M. J. Cich, and D. M. Mittleman. “A Spatial Light Modulator for Terahertz Beams.” Applied Physics Letters, **94**(21):213511 (2009).

[21] Y. He, H. Liang, R. Luo, M. Li, and Q. Lin. “Dispersion Engineered High Quality Lithium Niobate Microring Resonators.” Optics Express, **26**(13):16315 (2018).

[22] A. Shams-Ansari, G. Huang, L. He, Z. Li, J. Holzgrafe, M. Jankowski, M. Churaev, P. Kharel, R. Cheng, D. Zhu, N. Sinclair, B. Desiatov, M. Zhang, T. J. Kippenberg, and M. Lončar. “Reduced Material Loss in Thin-Film Lithium Niobate Waveguides.” APL Photonics, **7**(8):081301 (2022).

[23] X. Wu, C. Zhou, W. R. Huang, F. Ahr, and F. X. Kärtner. “Temperature Dependent Refractive Index and Absorption Coefficient of Congruent Lithium Niobate Crystals in the Terahertz Range.” Optics express, **23**(23):29729–29737 (2015).

[24] U. Deva and C. Saha. “Gain Enhancement of Photoconductive THz Antenna Using Conical GaAs Horn and Si Lens.” In “2016 International Symposium on Antennas and Propagation (APSYM),” pages 1–3. IEEE, Cochin, India (2016).

[25] G. Lu, R. Zhao, H. Yin, Z. Xiao, and J. Zhang. “Study of the Super Directive THz Photoconductivity Antenna.” Plasmonics, **16**(3):677–685 (2021).

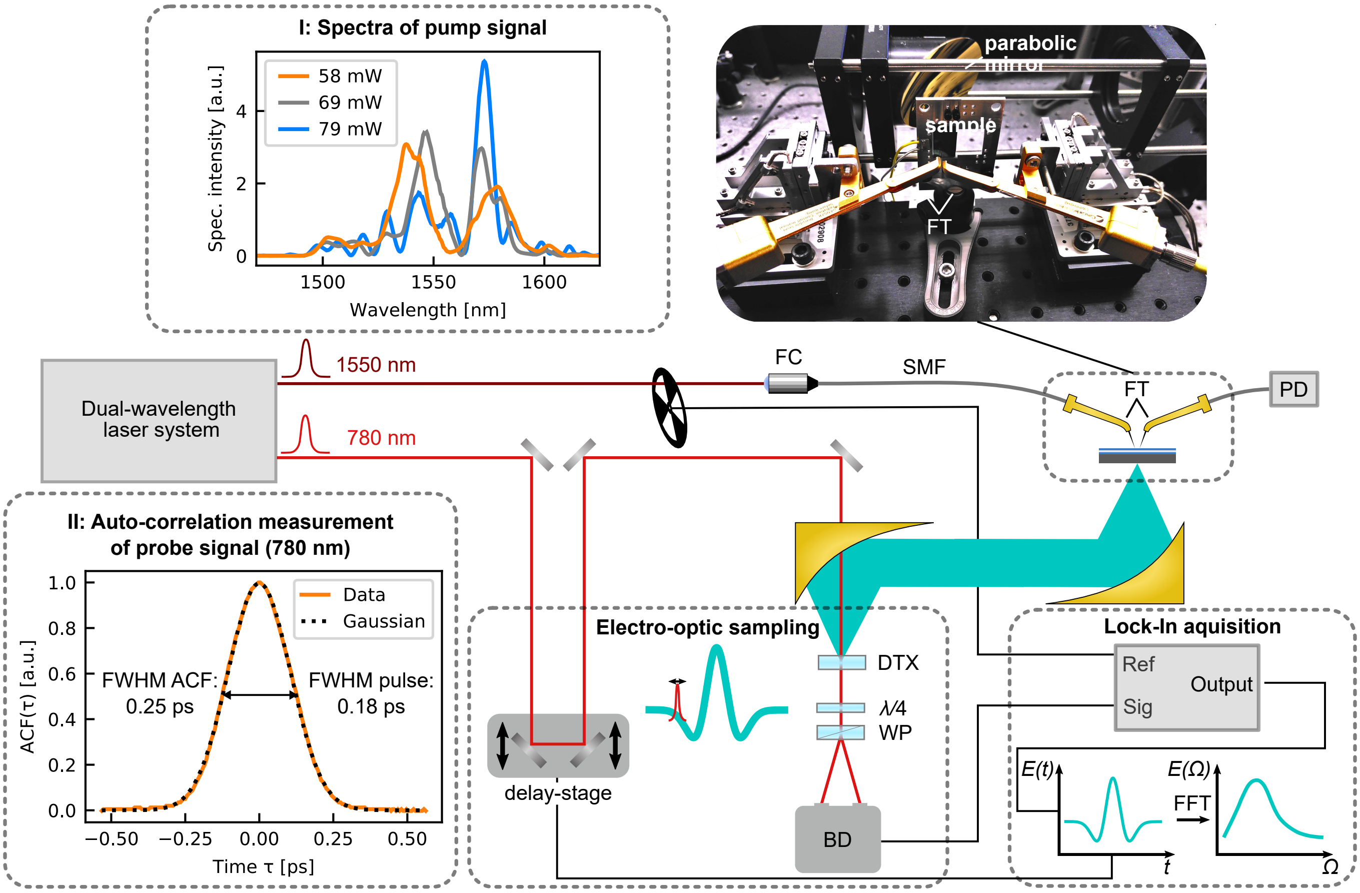

Supplementary Fig. 13: Dual wavelength time-domain THz spectroscopy. Both signals – the pump at 1560 nm (dark red) and the probe beam at 780 nm (light red) – originate from one laser System provided by MenloSystem. A fiber-collimator (FC) couples the pump pulse into a single mode fiber (SMF) guiding the signal via a cleaved fiber tip (FT) on chip. Panel I shows the spectra the of pump signal changing for different averaged powers due to nonlinear effects inside the fiber. A second fiber tip collects the remaining 1560 nm signal transmitted through the device to monitor its power on a photo-diode (PD). The photographic picture (top right corner) shows the sample in front of the collecting parabolic mirror and the piezo-stages positioning the fiber-tips to the grating on-chip grating couplers. The THz radiation (turquoise) emitted by the LN-chip is collected and focused into the zinc telluride crystal by a pair of parabolic mirrors. The synchronized fs-probe pulses (autocorrelation measurement plotted in panel II) are spatially and temporally overlaid with the THz pulses whereby a delay stage enables to control the time-delay between the two signals. The combination of a $\lambda/4$, Wolliston prism (WP) and a balanced detector (BD) enables an ellipsometric measurement of the change in the probe polarization introduced by the THz electric field. A chopper in the pump beam path delivers a reference signal for a lock-in amplifier measuring the electric THz field depending on the time-delay set by the delay stage (bottom right).

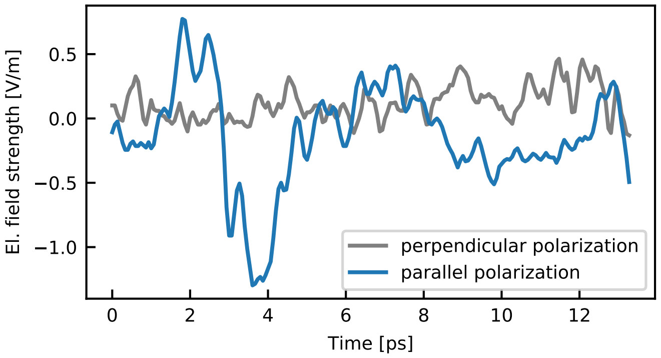

Supplementary Fig. 14: Polarisation properties of emitted THz radiation. Electric field emitted by two parallel bow-tie antennas polarized parallel (blue) and perpendicular (grey) to the antenna elongation.

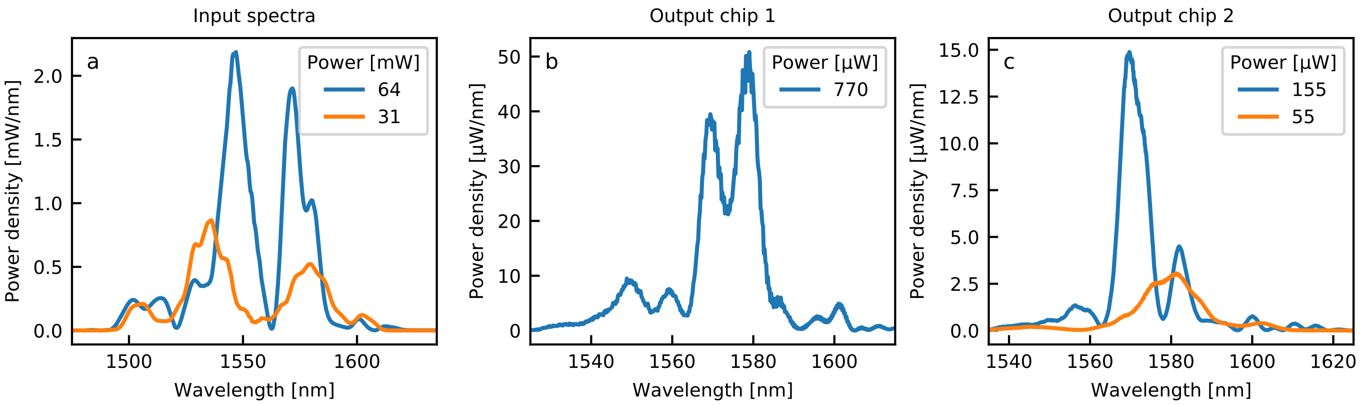

Supplementary Fig. 15: Pump power coupling to TFLN chip a: Spectral power density of the pump signal before it is coupled into the LN waveguides for the two different power configurations used in the current work. **b**: Spectrum of the near-infrared signal out-coupled from first-generation sample. The input power measured 64 mW. **c**: Spectral density of the pump pulses after the second-generation sample for 64 mW (blue) and 31 mW (orange) of input power.

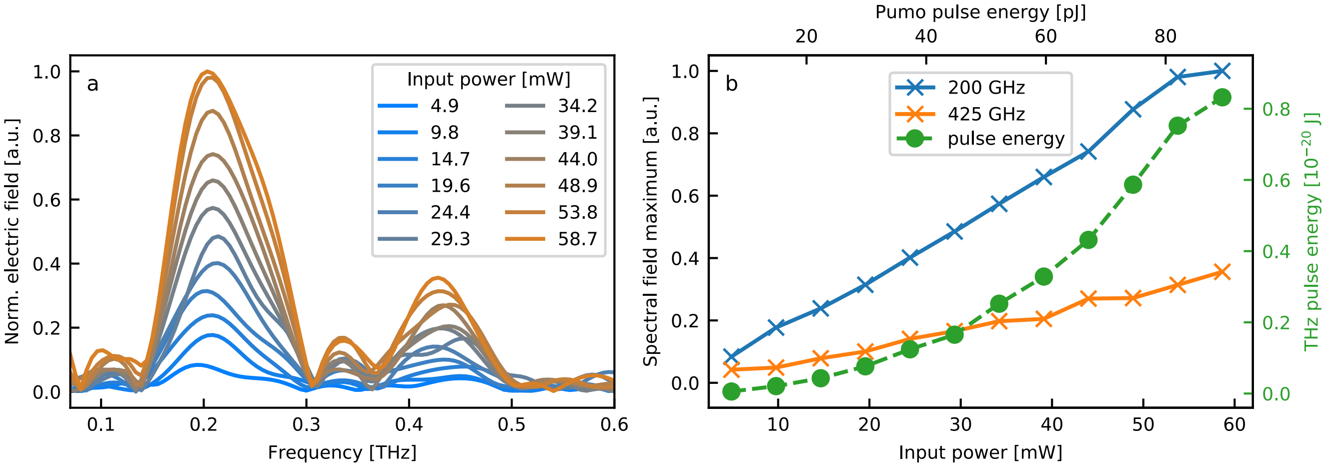

Supplementary Fig. 16: Pump power dependence of THz emission. a: Spectral electric field strength emitted by a 3-antenna array with varying pump power. **b**: In blue and orange (left axis) the peak electric field main and side peak in the spectrum plotted depending on the input power (lower axis) and the corresponding on-chip pulse energy (upper axis). The generated THz pulse energy determined according to the measured time-traces is plotted in green (right axis).

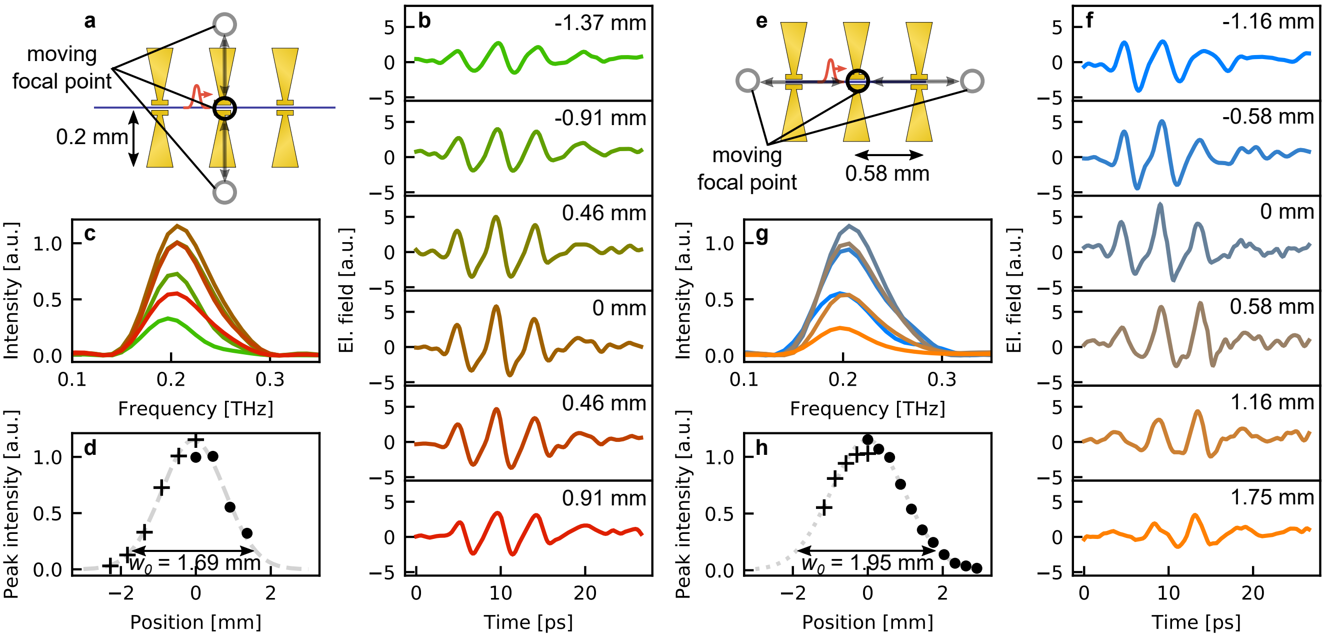

Supplementary Fig. 17: Measured farfield pattern of antenna emitter. **a** and **e**: Schematic illustration of the investigated device and the two direction the focal point is moved. **b** and **f**: Temporal electric field measured at different exemplary positions of the sample. The position of the center of the device with respect to the focal point in the direction perpendicular (b) and parallel (f) to the waveguide is indicated in each plot. **c** and **g**: Spectral intensity corresponding to the measured time-traces (b and f). **d** and **h**: Spectral peak intensity depending on the position of the sample fitted with a Gaussian function. For the perpendicular direction a focal beam diameter of 1.69 mm is determined, while in the direction parallel to the antenna array the diameter measures 1.95 mm. The points indicated by crosses indicate the measuring series moving in negative direction, while for the circles the sample was moved in positive direction.
